# Health and health behaviours in adolescence as predictors of education and socioeconomic status in adulthood – a longitudinal study

**DOI:** 10.1186/s12889-024-18668-7

**Published:** 2024-04-26

**Authors:** Leena K. Koivusilta, Paulyn Jean Acacio-Claro, Ville M. Mattila, Arja H. Rimpelä

**Affiliations:** 1 Department of Social Research, Faculty of Social Sciences, University of Turku, 20014 Turku, Finland; 2 Department of Health Policy and Administration, College of Public Health, University of the Philippines Manila, and Unit of Health Sciences, Faculty of Social Sciences, Tampere University, Tampere, Finland; 3 Faculty of Medicine and Health Technology, Tampere University, Tampere, Finland; 4 Department of Orthopaedics and Traumatology, Tampere University Hospital Tampere, Tampere, Finland; 5 Faculty of Social Sciences, Unit of Health Sciences, Tampere University, 33014 Tampere, Finland; 6 Department of Adolescent Psychiatry, Tampere University Hospital, P.O. Box 2000, 33521 Tampere, Finland

**Keywords:** Health, Health behaviours, Adolescence, Education level, Socioeconomic status, SES, Health selection, Social causation, Longitudinal study

## Abstract

**Background:**

The positive association of health with education level and socioeconomic status (SES) is well-established. Two theoretical frameworks have been delineated to understand main mechanisms leading to socioeconomic health inequalities: social causation and health selection but how these work in adolescence is poorly known. We studied if adolescent health and health behaviours predict higher education and higher SES in adulthood and if family background and school performance in adolescence explain these associations.

**Methods:**

Surveys on health and health behaviours were sent to representative samples of 12–18-year-old Finns in 1981–1997 every second year (response rate 77.8%, *N* = 55,682). The survey data were linked with the respondents’ and their parents’ socioeconomic data from the Finnish national registries. Both latent variables, namely, health (perceived health, health complaints, chronic disease), health-compromising behaviours (smoking status, drunkenness frequency), and family background (parents’ occupation-based SES, education, family type) and variables directly measuring health-enhancing behaviours (toothbrushing, physical activity) and school performance were used to predict higher education and higher occupation-based SES at age 34. Logistic regression analysis and structural equation models (SEM) were used.

**Results:**

In logistic regression analyses, good health, health-enhancing behaviours, and lack of health-compromising behaviours were related to higher education and SES, also after controlling for family background and school performance. In the SEM analyses, good health, health-enhancing behaviours, and lack of health-compromising behaviours directly predicted higher SES and higher education, although the standardised coefficients were low (from 0.034 to 0.12). In all models, health, lack of health-compromising behaviours, and health-enhancing behaviours predicted school performance, which in turn, predicted the outcomes, suggesting indirect routes to these. Good socioeconomic prospects in terms of family background predicted good health, healthy behaviours, and good school performance in adolescence and higher SES and higher education in adulthood.

**Conclusion:**

Health and health behaviours in adolescence predicted education and SES in adulthood. Even though the relationships were modest, they support the health selection hypotheses and emphasise the importance of adolescence for health inequalities during the life-course. Health and health behaviours were strongly associated with school performance and family background which together modified the paths from health and health behaviours to the outcomes.

**Supplementary Information:**

The online version contains supplementary material available at 10.1186/s12889-024-18668-7.

## Background

The relationship between health and socioeconomic status (SES) is well-established; poor health is associated with lower SES [1, 2]. SES covers a wide range of economic, social, and cultural resources, unevenly distributed in society. These resources people can use to build their health [3]. Two main theoretical frameworks have been delineated to understand the mechanisms leading to socioeconomic health inequalities. The model of social causation states that a person’s SES influences health. The model of health selection assumes that it is health which, at various stages of life, influences SES; good health helps a person in moving up in the system of social status hierarchies while for people with poor health, the route is opposite [4]. Of the two models, health selection has been considered less important [5] or with less evidence supporting it [3, 6–11], even though some studies have supported it [12–16]. Differences in health indicators, age of population, follow-up period, statistical methods, and socio-historical context can explain different results and explanatory power of the models [3, 5, 10, 17, 18]. Little is known if these two models function in adolescence or which mechanisms can be found at this stage of life which then lead to socioeconomic health differences in adolescence.

Already in 1997, West [19] presented a hypothesis of equalisation of social class health differences in youth (except for severe chronic illness). Later, West and Sweeting [20] showed that the association between SES and health is weak in adolescence and partly dependent on the used health measures. Adolescence is a period of life with physical and psychological growth, a transition from childhood to adulthood with its developmental tasks. Dependence from the childhood family decreases and the role of peers and external influences of the environment increases [19]. In childhood and adolescence, SES means family background while one’s own educational career and adult SES start to formulate through academic achievement and the chosen educational path [21]. There is evidence of, e.g., the association of academic achievement and health [22] which may be an early sign of socioeconomic health differences. Secondly, serious health effects of risky behaviours adopted in adolescence, like smoking of drinking, can be seen only after several years or even decades. These behaviours are more common among those children whose academic achievement is poor [23]. This shows another potential way to adulthood socioeconomic health differences during adolescence.

Equal opportunities for learning, high-quality teaching, or higher education are not available for every child and can be restricted by the child’s health. Poor health may set obstacles for education, which then leads to lower education in adulthood [7, 24–29]. Poor health may cause school absences, weak school engagement, repetition of grades [25, 26], or school dropout [16] and increase the risk for anxiety and depression [30–32]. Further, it may negatively shape a person’s self-image and attitude towards the future [33]. Mental health problems have had a stronger impact on school performance and career choice [7, 16], while the impact of somatic diseases have varied according to the condition [34]. In a Finnish study, a chronic disease in adolescence slightly weakened school performance, but its role as a predictor of educational path was small [3]. When considering adult SES, educational qualifications largely influence opportunities in the labour market, economic resources, and the probability of safe working conditions [35].

In addition to health, the health selection mechanisms in adolescence are present in the adoption of health behaviours [36]. Children with health-enhancing behavioural patterns do better at school and obtain higher educational qualifications [37–41]. Risky health behaviours are more common among children with poor school performance, who leave school early and who do not choose an academic path but, instead, vocational school [39]. This means decreasing chances for high education and an increasing probability of later health problems among those with poor school performance [9, 38]. Risky health behaviours are also a contributory factor in less smooth transitions between the basic and secondary education [42] and in school drop-out, and they are related to an increased risk for NEET (not in employment, education, or training) and unemployment in adulthood [43, 44].

The societal context where families make decisions of their child’s education brings along an element of social causation in the selection process. The differentiation of educational careers is strongly based on family SES. Parents’ high SES is associated with the child’s good school achievement [45, 46]. It also predicts the child’s own SES when reaching adulthood [47–49]. One more feature influential for the children’s educational careers is family type. It has been shown that single-parent families more often than two-parent families have poorer socio-economic circumstances and problems which may negatively affect the children’s wellbeing [50–53]. There is evidence about poorer cognitive development and academic achievement among children from single-parent families [54]. Recently, the impact of cumulative socioeconomic and family-related disadvantage on children’s socioeconomic careers has been emphasized [55].

The setting of our study is Finland, which has a long tradition of a comprehensive school system aimed to give equal educational opportunities for each child regardless of gender, social background, or place of residence. Finnish basic education (grades 1–9) is formally uniform, including no official tracking or ability grouping of students until upper secondary education. Differences in students’ academic performance between schools have been small, when compared internationally [56]. An important educational transition takes place after the 9th grade, at the age of 16. Based on school performance and own interests, the adolescent continues to academic or the vocational track in the upper secondary education or discontinues education. Socio-politically and individually, it is considered desirable if a young person chooses the academic path, because this is the main route to tertiary level education and, on average, to a steadier professional career and higher SES. Those who complete vocational education may have a smooth transition from education to work, but they may not be equipped with resources applicable in the rapidly changing labour market of the information society [57].

The models of health selection and social causation are not mutually exclusive, but chainlike and intertwined processes operate during a person’s life-course leading to health inequalities [12, 18, 58–60]. In this study, we aim to add understanding of how the processes of health selection operate between adolescence and adulthood, and if these processes operate independently from the processes of social causation. More specifically, we study

- if adolescent health and health behaviours predict higher education and higher SES in adulthood; and
- if family background and school performance in adolescence explain the paths from adolescent health and health behaviours to higher education and higher SES in adulthood.

**Fig. 1:**
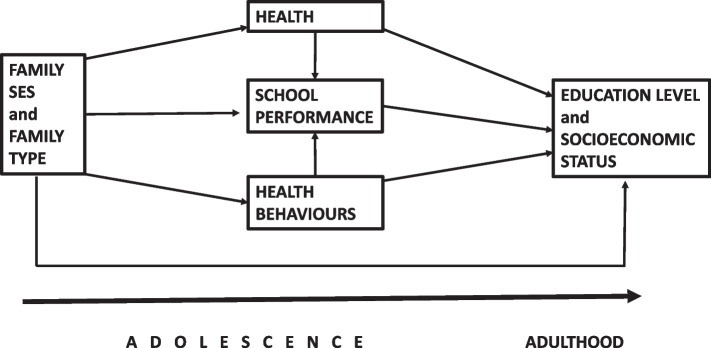
Theoretical model for the associations between adolescent health and health behaviours and adulthood education and SES

A theoretical model for the research is presented in Fig. 1, which shows the hypothesized pathways from adolescence to adulthood.

## Methods

### Study design and population

A nationally representative sample of 12-, 14-, 16-, and 18-year-old Finns was drawn from the Population Registry Centre, Finland in February 1981. A self-administered questionnaire on health and health behaviours was sent by post to the home addresses of the sample adolescents. The non-respondents received two re-inquiries. Every second year from 1985, a corresponding sample was drawn from the Population Registry Centre and a corresponding questionnaire was mailed to them. We use here data from 1981 to 1997. The overall response rate was 77.8% (*N* = 55682), 72.3% (*N* = 26042) for boys and 84.8% (*N* = 29640) for girls. The survey data were individually linked with the respondents’ and their parents’ sociodemographic data, obtained from national registries. The registry data on socioeconomic circumstances had been obtained from national censuses conducted every fifth year until 1995 and from on-line registry data on a yearly basis from 2000 onwards. Statistics Finland linked the survey data with the data from the national registries concerning both the survey respondents and their parents.

For a survey respondent, the follow-up ended when the respondent reached the age 34 years meaning an individual follow-up time of 16 to 22 years. This was the maximum age that all survey respondents could reach before the end of the follow-up on 31st December 2018. Data on mortality by Statistics Finland were used to exclude the dead from the analysis (*N* = 750).

### Outcome variables (the survey respondent; achieved by the age of 34 years)

The survey respondents’ socioeconomic status in the society was measured by years of education and by occupation-based socio-economic status.

#### Education

The variable from the education registry from Statistics Finland was coded binary: 1 = higher (over 12 years of education), 0 = lower (at most 12 years). Statistics Finland uses 12 years as a cut-point because that means higher middle-level or tertiary education.

#### Occupation-based socio-economic status (SES)

The variable was based on the occupation in the registries of Statistics Finland and binary-coded: 1 = higher (upper or lower white-collar, entrepreneur), 0 = lower (blue-collar, pensioner, student, in military service, unknown). This variable had 5786 missing values (10.4%). These were excluded from the analysis.

### Explanatory variables (adolescence)

#### Health

Three questions on health were presented. *Perceived health* at the time of inquiry: very good, good, average, poor (rather or very poor). *Daily stress symptoms* composed a summary index of eight health complaints (stomach aches, tension or nervousness, irritability or outbursts of anger, trouble falling asleep or waking at night, headache, trembling of hands, feeling tired or weak, feeling dizzy) and classified as follows: none, one, 2, 3–8. *A chronic disease, injury or disability* that restricted daily activities was classified: no, yes.

#### Health behaviours

Two health-compromising behaviours were considered. *Smoking status* variable was defined differently in age groups to reflect the process of smoking initiation. The 12-year-olds were defined as smokers if they had smoked more than two cigarettes and, 14-year-olds, if they had smoked more than 50 cigarettes in their lifetime. Among the 16–18-year-olds, smokers were those who smoked daily. In each age group, others were defined as non-smokers. *Drunkenness frequency* described the respondents’ frequency of drunkenness: never, at most 1–2 times a month, once a week or more often.

Behaviours defined as health-enhancing were the following. *Physical exercise* was measured by reported shortness of breath or sweating during exercise with the categories: vigorous, to some extent, no (a little/not at all/does not exercise). Categorie*s* for* tooth brushing habit* were as follows: brushes several times a day, once a day, and 1–5 times a week or less.

#### School performance

School performance was measured by asking the respondents’ self-assessments of the latest end-of-term school report compared with the class average. The categories were: much better, slightly better, average, slightly poorer, much poorer. This was used for 12–14-year-olds, who were all in comprehensive school, as a proxy for their later educational track after the comprehensive school (academic vs. vocational). For 16–18-year-olds, we combined information on performance and the school type (academic vs. vocational). We formed a variable over the ages, which indicated probabilities of reaching higher educational levels in adulthood: highest (ages 12–14: school performance much better than average; 16–18: academic track, much better than average), second highest (12–14: better than average; 16–18: academic and better than average/average or vocational and much better/better than average), second lowest (12–14: average; 16–18: academic and slightly poorer than average or vocational and average/slightly poorer than average), lowest (12–14: slightly/much poorer than average; 16–18: academic and much poorer than average or vocational and much poorer than average/not at school).

#### Family background

The parents’ data, nearest to the child age of 15 years, was extracted from the national registries and linked with the survey data, to measure the child’s socioeconomic status during adolescence.

Similarly, to the outcome variables, two indicators, education level and SES, based on the classifications of Statistics Finland were used for parents’ SES. Data on father and mother were combined. If the categories for mother and father were different, the higher one was chosen. If one was missing, the other one was used. Exact age information was not available because censuses before 2000 were collected only every 5th year. Data from parents who died before the respondents’ survey were recorded as missing.

The categories of parental education level in the original variable were the following: high (over 12 years in education), middle (10–12 years), low (9 years or less). The combined *parents’ education* variable was formed as follows: both high, either one high, either one middle, both low. The original SES had categories: upper white-collar or entrepreneur, lower white-collar, agricultural employer or entrepreneur, blue-collar, pensioners, other (pensioners, students, other, unknown). The combined *parents’ SES* variable was formed as follows: both parents upper white-collar, either one upper white-collar, either one lower white-collar, either one blue-collar or both unknown. Entrepreneurs were included in the white-collar categories.

*Family type* was categorized as follows: both parents, other. The variable was taken from the surveys.

The characteristics of respondents are shown in Additional file 1 (see Additional file 1).

#### Statistical analysis

Both binary logistic regression analysis and structural equation modelling (SEM) were used to study if the data followed the theoretical model (Fig. 1). First, the univariate associations of the predictor variables with each outcome variable were studied. Second, each one of the health and health behaviour variables were treated separately in multivariable logistic analyses to study whether their associations with the outcomes were accounted for by school performance and family background. The models were also adjusted for study year and gender. Listwise deletion was used to handle missing data. Odds ratios (OR) and 95% confidence intervals (95% CI) were reported.

Third, in SEM, linear probability models were fitted to study how health and health behaviour in adolescence predicted the probability of higher education and higher SES in adulthood. Latent variables were formed from the measured ones via confirmatory factor analysis. The latent variable “health” was measured by perceived health, chronic disease, and health complaints daily. The latent variable “health-compromising behaviours” included smoking status and drunkenness frequency, while health-enhancing behaviours were measured directly by physical exercise and tooth brushing habit. The method maximum likelihood with missing values was used. The standardised regression coefficients for the measurement models, estimating paths formed from latent variables to the observed variables, are shown only in the text. For model fit, the RMSEA (root mean square error of approximation), CFI (comparative fit index), and TLI (Tucker-Lewis index) were used [61]. Covariances between the outcomes were allowed and so were those between the measured variables if they reasonably improved the model fit. In all analyses, statistical significance was set at *p* < 0.01 to ensure detecting non-spurious associations between the variables [62]. Logistic regression analyses were done using SPSS version 27 [63] and Stata, version 17 was used for SEM [64]. All analyses took place in Statistics Finland's Fiona remote operating system, with the methods available there, ensuring analyses were done in a controlled environment [65].

## Results

### Associations of adolescent health and health behaviours with education in adulthood

In gender-specific univariate analyses, all variables measuring adolescent health and health behaviours were statistically associated with high education in adulthood (Table 1). Better health, enhancing health behaviours and not having health-compromising behaviours systematically predicted higher education. However, compared to the health and health behaviours, school performance in adolescence and parents’ SES and education were stronger predictors of the outcome. Differences between boys and girls were minor. When adjusted for study year, gender and school performance, the associations of all health and health behaviour variables with education in adulthood weakened but stayed statistically significant. Adjusting further for family background, the odds ratios changed only a little. These adjusted analyses were not done separately for the genders, because no remarkable gender differences were observed in the univariate analyses.

**Table 1:** ORs^a^ and 95% CI for higher education in adulthood, according to explanatory variablesin adolescence

Explanatory variables in adolescence	Higher education in adulthood			
	Boys	Girl	Adjusted for study year, gender and school performance	Adjusted for previous variables and family background
**Health**				
Perceived health				
Very good	**1.8 (1.4–2.2)**	**2.0 (1.7–2.4)**	**1.3 (1.2–1.6)**	**1.3 (1.2–1.6)**
Good	**1.7 (1.3–2.1)**	**1.7 (1.4–2.0)**	**1.2 (1.1–1.5)**	**1.3 (1.1–1.5)**
Average	1.2 (1.0–1.5)	1.2 (1.0–1.5)	1.2 (1.0–1.3)	1.2 (1.0–1.3)
Poor	1.0	1.0	1.0	1.0
Chronic disease				
No	**1.1 (1.0–1.2)**	**1.2 (1.1–1.3)**	**1.2 (1.1–1.2)**	**1.1 (1.1–1.2)**
Yes	1.0	1.0	1.0	1.0
Health complaints daily				
None	**2.3 (1.8–2.9)**	**1.9 (1.7–2.1)**	**1.6 (1.4–1.8)**	**1.4 (1.4–1.7)**
One	**1.6 (1.3–2.1)**	**1.5 (1.3–1.7)**	**1.3 (1.1–1.5)**	**1.1(1.1–1.4)**
2	1.5 (1.1–1.9)	1.2 (1.0–1.4**)**	1.1 (1.0–1.3)	1.0 (1.0–1.3)
3 to 8	1.0	1.0	1.0	1.0
**Health-compromising behaviours**				
Smoking status				
Non-smoker	**2.7 (2.5–2.8)**	**2.4 (2.3–2.5)**	**1.8 (1.7–1.9)**	**1.7 (1.6–1.8)**
Smoker	1.0	1.0	1.0	1.0
Drunkenness frequency				
Never	**2.6 (2.2–3.0)**	**2.6 (2.2–3.0)**	**1.7 (1.5–1.9)**	**1.6 (1.4–1.58)**
At most 1–2 times a month	**2.3 (2.0–2.6)**	**2.1 (1.8–2.5)**	**1.6 (1.4–1.8)**	**1.5 (1.4–1.7)**
Once a week	1.0	1.0	1.0	1.0
**Health-enhancing behaviours**				
Physical exercise				
Vigorous	**2.2 (2.2–2.5)**	**1.9 (1.7–2.0)**	**1.5 (1.4–1.6)**	**1.5 (1.4–1.5)**
To some extent	**1.5 (1.5–1.7)**	**1.5 (1.4–1.6)**	**1.3 (1.2–1.3)**	**1.3 (1.3–1.4)**
No	1.0	1.0	1.0	1.0
Tooth brushing habit				
Several times a day	**3.2 (3.0–3.5)**	**2.2 (2.0–2.5)**	**1.9 (1.8–2.1)**	**1.9 (1.8–2.1)**
About once a day	**2.2 (2.0–2.3)**	**1.7 (1.5–1.9)**	**1.6 (1.5–1.7)**	**1.6 (1.5–1.7)**
At most 1–5-times a week	1.0	1.0	1.0	1.0
**School performance**				
Highest	**15.6 (13.4–18.1)**	**14.0 (12.2–16.0)**		
Second highest	**11.6 (10.2–13.3)**	**9.7 (8.5–10.9)**		
Second lowest	**2.7 (2.4–3.1)**	**3.0 (2.7–3.5)**		
Lowest	1.0	1.0		
**Family background**				
Parents’ education				
Both high	**5.9 (5.2–6.7)**	**3.4 (3.0–3.9)**		
One high	**4.7 (4.3–5.2)**	**2.7 (2.5–2.9)**		
One middle	**1.9 (1.7–2.0)**	**1.5 (1.4–1.6)**		
Both low	1.0	1.0		
Parents’ SES				
Both upper white-collar	**3.1 (2.8–3.3)**	**2.6 (2.4–2.8)**		
One upper white-collar	**2.7 (2.5–2.9)**	**2.5 (2.0–2.3)**		
One lower white-collar	**2.1 (2.0–2.3)**	**1.9 (1.7–1.9)**		
Blue-collar or unknown	1.0	1.0		
Family type				
Both parents	**2.0 (1.8–2.1)**	**1.9 (1.8–2.1)**		
Other	1.0	1.0		

^a^Statistically significant associations are shown in bold

### Associations of adolescent health and health behaviours with SES in adulthood

In gender-specific univariate analyses, all health and health behaviour variables in adolescence, except chronic disease in boys, predicted higher SES in adulthood (Table 2). School performance was the strongest predictor, but compared with the results in Table 1, its predictive power for adulthood SES was smaller than for education in adulthood. Parents’ education and SES were stronger predictors than health and health behaviours. The associations followed a similar pattern for both genders. When adjusted for study year, gender and school performance in adolescence, the associations weakened but stayed mainly statistically significant. Only minor changes in odds ratios were seen when a further adjustment for family background was performed.

**Table 2:** ORs^a^ and 95% CI for higher SES in adulthood, according to explanatory variables in adolescence

Explanatory variables in adolescence	Higher SES in adulthood				
	Boys		Girl	Adjusted for study year, gender and school performance	Adjusted for previous variables and family background
**Health**					
Perceived health					
Very good	**1.8 (1.4–2.2)**		**2.0 (1.7–2.4)**	**1.5 (1.3–1.7)**	**1.4 (1.3–1.7)**
Good	**1.6 (1.3–1.9)**		**1.7 (1.4–2.0)**	**1.4 (1.2–1.6)**	**1.3 (1.2–1.6)**
Average	1.2 (1.0–1.5)		1.2 (1.0–1.5)	1.2 (1.0–1.4)	1.2 (1.0–1.4)
Poor	1.0		1.0	1.0	1.0
Chronic disease					
No	1.2 (1.0–1.2)		**1.3 (1.2–1.4)**	**1.1 (1.1–1.2)**	**1.1 (1.1–1.2)**
Yes	1.0		1.0	1.0	1.0
Health complaints daily					
None	**1.7 (1.4–2.1)**		**1.9 (1.7–2.12**	**1.6 (1.4–1.8)**	**1.5 (1.3–1.7)**
One	1.3 (1.1–1.6)		**1.5 (1.3–1.8)**	**1.3 (1.2–1.5)**	**1.3 (1.1–1.5)**
2	1.1 (0.9–1.5)		**1.2 (1.1–1.5)**	1.1 (1.0–1.3)	1.1 (1.0–1.3)
3 to 8	1.0		1.0	1.0	1.0
**Health-compromising behaviours**					
Smoking status					
Non-smoker	**2.0 (1.9–2.1)**		**1.7 (1.6–1.8)**	**1.4 (1.3–1.5)**	**1.3 (1.3–1.4)**
Smoker	1.0		1.0	1.0	1.0
Drunkenness frequency					
Never	**1.9 (1.7–2.2)**		**1.7 (1.5–2.0)**	**1.3 (1.2–1.5)**	**1.3 (1.1–1.4)**
At most 1–2 times a month	**1.9 (1.6–2.1)**		**1.7 (1.5–2.0)**	**1.4 (1.3–1.6)**	**1.4 (1.2–1.5)**
Once a week	1.0		1.0	1.0	1.0
**Health-enhancing behaviours**					
Physical exercise					
Vigorous	**2.1 (2.0–2.2)**		**1.8 (1.6–1.9)**	**1.6 (1.5–1.7)**	**1.5 (1.4–1.6)**
To some extent	**1.4 (1.3–1.5)**		**1.4 (1.4–1.5)**	**1.3 (1.2–1.3)**	**1.2 (1.2–1.3)**
No	1.0		1.0	1.0	1.0
Tooth brushing habit					
Several times a day	**2.7 (2.5–2.9)**		**2.1 (1.9–2.3)**	**1.8 (1.7–2.0)**	**1.7 (1.6–1.8)**
About once a day	**1.8 (1.7–2.0)**		**1.6 (1.5–1.8)**	**1.5 (1.4–1.6)**	**1.4 (1.4–1.5)**
At most 1–5-times a week	1.0		1.0	1.0	1.0
**School performance**					
Highest		**7.7 (6.7–8.7)**	**4.4 (3.9–5.0)**		
Second highest		**4.7 (4.3–5.3)**	**2.8 (2.6–3.1)**		
Second lowest		**1.4 (1.3–1.6)**	**1.1 (1.1–1.4)**		
Lowest		1.0	1.0		
**Family background**					
Parents’ education					
Both high		**5.0 (4.3–5.8)**	**2.6 (2.2–3.0)**		
One high		**4.0 (3.6–4.4)**	**2.2 (2.0–2.5)**		
One middle		**1.6 (1.5–1.7)**	**1.4 (1.3–1.5)**		
Both low		1.0	1.0		
Parents’ SES					
Both upper white-collar		**3.5 (3.2–3.8)**	**2.1 (1.9–2.3)**		
One upper white-collar		**2.9 (2.7–3.1)**	**2.0 (1.9–2.2)**		
One lower white-collar		**2.1 (1.9–2.2)**	**1.9 (1.7–2.0)**		
Blue-collar/ both unknown		1.0	1.0		
Family type					
Both parents		**1.6 (1.5–1.8)**	**1.6 (1.5–1.7)**		
Other		1.0	1.0		

^a^Statistically significant associations are shown in bold

### Linear probability models (SEM) for the associations between health, health behaviours, school performance and the outcomes

#### Health

**Fig. 2:**
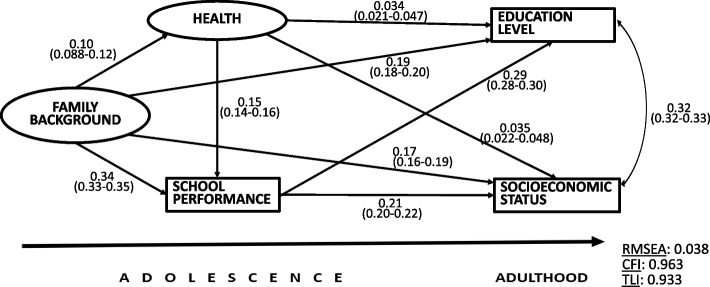
The SEM analysis for health. Standardized regression coefficients, 95% confidence intervals and model fit indicators

Figure 2 shows the SEM model including two latent variables, health and family background. In the measurement model of the latent variable Health, the standardized coefficients were 0.6 for the observed variable perceived health, 0.25 for chronic disease, and 0.4 for daily stress symptoms. In the measurement model of the latent variable Family background, the coefficients were 0.65 for parents’ education, 0.62 for parents’ SES, and 0.2 for family type. Larger coefficients reflected greater strength of the relationship with the latent variable. The covariance between the outcome variables was 0.3.

The standardised coefficients of the paths from Health to Education and SES in adulthood were small (0.034 and 0.035) but showed that better health was related to higher SES and education in adulthood. Good health predicted better school performance, which in turn, predicted higher SES and education in adulthood. Good social prospects in terms of family background predicted better health, better school performance and higher education and SES in adulthood. All coefficients were statistically significant (*p < *0.001) and the short confidence intervals showed their high precision in estimating the associations.

#### Health-compromising behaviours

**Fig. 3:**
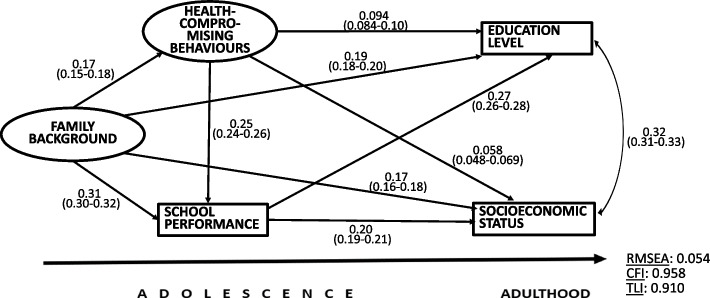
The SEM analysis for health-compromising behaviours. Standardized regression coefficients, 95% confidence intervals and model fit indicators

Figure 3 shows the SEM model for health-compromising behaviours. In the measurement model of the latent variable Health-compromising behaviours, the standardized coefficients of the observed variables were 0.86 for smoking status and 0.46 for drunkenness frequency. The coefficients for the measurement model of Family background were equal to those in the previous model presented for Health. The covariance between the outcomes was 0.32.

The standardised coefficients for the paths from health-compromising behaviours to the outcomes were rather small (0.094 and 0.058) but positive showing that the lack of these behaviours predicted high education and SES in adulthood. The coefficients from health-compromising behaviours to school performance meant that the lack of these behaviours predicted better school performance, which in turn was positively related with the outcomes. High family background directly predicted higher education and SES. It was also associated with good school performance and the lack of health-compromising behaviours. All coefficients were statistically significant (*p* < 0.001) and the short confidence intervals showed their high precision in estimating the associations.

#### Health-enhancing behaviours

**Fig. 4:**
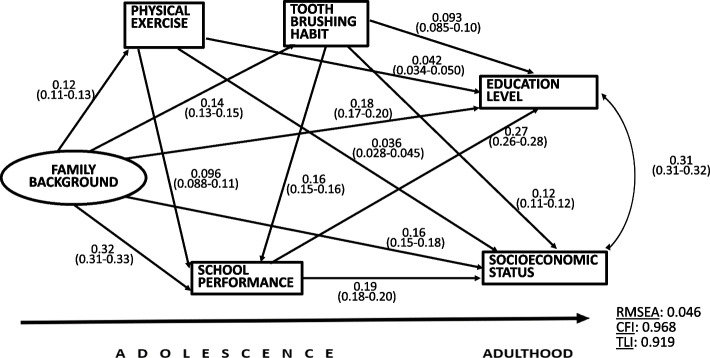
The SEM analysis for health-enhancing behaviours. Standardized regression coefficients, 95% confidence intervals and model fit indicators

Figure 4 shows the SEM model for health-enhancing behaviours, physical activity and tooth brushing habit. These were treated as observed variables, because a latent variable was not feasible due to small coefficients in the measurement model. The coefficients for the measurement model of Family background were equal to those in the previous models. The covariance between the outcome variables was 0.31.

Health-enhancing behaviours predicted higher SES and education in adulthood, but the relationships were not strong. The standardised coefficient of the path from physical activity to education in adulthood was 0.042 and to SES it was 0.036. The corresponding coefficients from tooth brushing habit were 0.093 and 0.12. Family background predicted both behaviours, school performance and both outcomes. Tooth brushing habit predicted school performance more strongly (0.16) than did physical activity (0.096), and school performance predicted both outcomes. All coefficients were statistically significant (*p* < 0.001) and the short confidence intervals showed their high precision in estimating the associations.

## Discussion

In our longitudinal study, better health, health-enhancing behaviours and not practicing health-compromising behaviours in adolescence predicted higher educational level and higher SES in adulthood, independently of family background and school performance. This finding emphasises the role of adolescence in the formation of health inequalities during the life-course. Even though the associations were modest, they support the health selection hypothesis [4]. Good school performance predicted higher education and SES in adulthood, and an indirect path from adolescent health and health behaviours through school performance was found, too. On the other hand, family background predicted adult outcomes directly so that adolescents from more educated and higher SES families more often than other young ended up in high education and SES. Family background predicted the outcomes also indirectly through school performance, health, and health behaviours. Thus, also the theory of social causation gets support.

Our results strengthen the earlier but somewhat weak evidence of the existence of the health selection model in the formation of health inequalities [12, 66]. Our long follow-up covered the critical years of transition from adolescence to adulthood, from the age of 12—18 years to the age of 34. Earlier studies with longer follow-up periods have shown, too, that health selection processes may take place particularly during the transition from adolescence to adulthood [67]. This means that low educated adults and adults in low SES groups have had more often a risky health behaviour pattern and more health problems already in adolescence, compared to adults in well-educated and high SES groups. Tracking of health behaviours from adolescence to adulthood is shown in many studies [68–70]. This means a higher risk for several non-communicable diseases in adulthood in the low SES group. Correspondingly, poor perceived health and mental health problems and many chronic diseases like asthma or diabetes, present in adolescence, are likely to continue to adulthood [71, 72].

The role of family background in the formation of education and SES in adulthood was strong. Our study could not go deeper into the mechanisms behind this association, but earlier literature gives some suggestions. Families with more economic resources tend to invest in the education of their children compared to families with fewer resources who may be more concerned with daily living expenses [66]. The direct association could also mean parents’ positive attitudes for high education, favourable social circumstances, and resources for the child to grow up, or parents’ professional networks to support their offspring’s career to maintain the social status and prevent their downward social mobility [73]. Parents’ favourable attitudes to education could explain, at least partly, the strong indirect association via school performance.

The SEM models showed the associations of family social background with health and health behaviours in adolescence. This points to the model of social causation, i.e., that a young person’s family SES causes differences in their health and development as has been suggested by earlier studies [66, 67, 74]. However, the associations were smaller compared to those with school achievement. If school achievement is regarded as an indicator of the young person’s future adult SES, our analysis also anticipates the re-emergence and strengthening of health inequalities in adulthood, as presented earlier by West [19].

Furthermore, if a child has health problems, educated or well-off parents can use their resources to prevent these from causing too much harm to school and learning [75]. Family backgrounds also influence the ways how health behaviours are adopted as part of the young people’s lifestyle. Health behaviours could reflect model learning or other mechanisms through which behaviours are transmitted intergenerationally and in peer groups. Smoking is an example of this [76, 77]. Norms concerning smoking habit differ between SES groups [78]. Likewise, the level of physical activity among children is positively connected with both family SES and parents’ exercise behaviour [79, 80]. Families also differ in their ways the parents monitor their children’s daily practices like those influencing oral health and alcohol use [81, 82].

As expected, school performance in adolescence was a strong predictor of education and SES in adulthood. School performance offered an indirect, mediated route from health and health behaviours to the adult outcomes: poor health and unhealthy behaviours predicted lower school performance which, in turn, predicted lower education and lower SES in adulthood. These indirect routes were stronger than the direct routes from health and health behaviours to the outcomes. Our data set was not able to reveal deeper the mechanisms leading from health and health behaviours to various levels of school performance but e.g., health-compromising behaviours are associated with poor schoolwork engagement and schoolwork difficulties [83], and the lack of some psychological resources such as self-efficacy [84]. Behaviours may be either reasons for school difficulties or their consequences. They may signalize difficulties in finding one’s place in the school community and among peers [20, 85]. Personal roles as members of peer groups as well as school type and smoking culture have been found to influence taking up the habit [86]. Health-compromising behaviours may indicate early identification with behaviour norms of social groups in which health promotion is not so important [87]. In further research, data on peer relationships and youth culture should be included, because they form a context important for both health, health behaviours and schooling [19, 88].

Among our three indicators of health, that of perceived health had the strongest weight in the measurement model in SEM. Each indicator predicted the outcomes when analysed separately, but the associations were the smallest for chronic disease. In another Finnish study, a long-term somatic illness weakened school success, but had a very weak impact on educational choices [45]. The findings are in line with those of West and Sweeting [20]. They found that, self-rated health, but not longstanding illness showed SES-related inequality among the young age groups.

Health behaviour is a multifaceted entity [89]. We used four key variables to measure it. They were grouped into health-compromising and health-enhancing ones. The classification of behaviours into these dimensions may be problematic. That’s why the entity of health-compromising variables was combined in one measurement model, but there was a need to treat the two health-enhancing variables separately to improve the model fit.

Our findings show a need to refine the health-selection model. In the direct selection, poor health as such decreases one’s possibilities for high education and social position. In the transition from adolescence to adulthood, this model seems too simple. Even if we found modest direct routes from adolescent self-reported health and health behaviours to education and social position in adulthood, the indirect or mediating routes via school performance were more important. And we cannot forget the role of family background in modifying school performance and health and health behaviours as well as their relationships. There seems to operate a complex process of an indirect health selection in which, during the transition from adolescence to adulthood, several factors and routes modify and complete the direct path and cause health inequality.

### Strengths and weaknesses of the study

The follow-up of our study was long, from the ages of 12–18 to the age of 34 years with large number of respondents with an excellent response rate. The follow-up time covered the critical years of adolescence in the transition to adulthood. The surveys were nationally representative, and the response rates were good. National registries offered a reliable source for education and SES indicators of the survey respondents and their parents, which can be considered much more reliable than children’s answers which are often the only available data source. The use of the statistical technique, SEM modelling, enabled us to study the importance of mediating factors and indirect pathways.

Some limitations can be noted. Survey answers were self-reported, and these always have random errors which, however, in large samples are become smaller. The reliability of the answers in our study has been shown good [90, 91]. The study covered the life course from the beginning of adolescence, i.e., from the age of 12 to adulthood. The surveys were based on repeated cross-sections of the cohorts, and hence, it was not possible to analyse the cause-and-effect relationships within the period of adolescence (from age 12 to age 18), but only between adolescence and adulthood. Thus, when testing the pathway from health/health behaviours to school performance, not a real causational association was described. Because poor health and unhealthy behaviours could also be regarded as indicators of poor school performance, it would have been equally possible to hypothesize arrows pointing to the opposite direction. We did not study this possibility, because due to computational difficulties, we decided to keep the models simple.

## Conclusions

The study showed that health and health behaviours in adolescence, both health-compromising and health-enhancing, predict SES in adulthood, when measured with education level and occupation-based SES. Even if the associations detected were modest, they gave support for the health selection model. Adolescent health and health behaviours were strongly connected with family background and school performance, too, and these together modified the paths towards socioeconomic positions in adulthood. This finding reminds of that the processes of health selection and the processes of social causation cannot be separated but they both work simultaneously during the life-course. Furthermore, our study emphasized adolescence as a particular stage of life where adult health inequalities arise. Success in school and decisions on educational track in adolescence are important in leading individuals towards their positions in the social structure of the society.

### Supplementary Information

**Supplementary Material 1. **Explanatory variables in adolescence (ages 12–18, 1981, 1985–1997) according to the outcomes in adulthood.

## Data Availability

Due to the existing Finnish legislation, sharing the data is not possible.

## Abbreviations

SES: Socioeconomic status
SEM: Standardised equation model
OR: Odds ratio
95% CI: 95% Confidence interval
