# Supplementary material for: Health and health behaviours in adolescence as predictors of education and socioeconomic status in adulthood – a longitudinal study
Source: BMC Public Health. 2024 Apr 26;24:1178. doi: 10.1186/s12889-024-18668-7 (PMC11055384; doi:10.1186/s12889-024-18668-7)
Supplement: Supplementary file 1 — Supplementary Material 1. Explanatory variables in adolescence (ages 12–18, 1981, 1985–1997) according to the outcomes in adulthood. [file 12889_2024_18668_MOESM1_ESM.docx]

Additional file 1. Explanatory variables in adolescence (ages 12-18, 1981, 1985-1997) according to the outcomes in adulthood

| Explanatory and confounding variables | Total population  N=55682^1^) | | Education in adulthood | | SES in adulthood | |
| --- | --- | --- | --- | --- | --- | --- |
|  |  |  | Higher  Total N=22393 | Lower  Total N=33289 | Higher  Total N=31529 | Lower  Total N=18367 |
|  | N | % | Row % | Row % | Row % | Row % |
| **Health** |  |  |  |  |  |  |
| Perceived health  Very good  Good  Average  Poorer | 18124  27663  8613  1066 | 32.7  49.9  15.5  1.9 | 42.5  41.1  34.0  29.7 | 57.5  58.9  66.0  70.3 | 65.2  64.0  57.9  53.0 | 34.8  36.0  42.1  47.0 |
| Chronic disease, injury or disability  No  Yes | 50752  4930 | 91.1  8.9 | 40.5  36.8 | 59.5  63.2 | 63.5  60.2 | 36.5  39.8 |
| Health complaints daily  None  One  2  3 to 8 | 44092  7374  2569  1647 | 79.2  13.2  4.6  3.0 | 41.5  37.2  33.8  29.9 | 58.5  62.8  66.2  70.1 | 64.1  61.5  58.0  54.3 | 35.9  38.5  42.0  45.7 |
| **Health behaviours** |  |  |  |  |  |  |
| Smoking status  Non-smoker  Smoker | 42012  13670 | 75.4  24.6 | 45.3  24.7 | 54.7  75.3 | 66.8  51.8 | 33.2  48.2 |
| Drunkenness frequency  Never  At most 1-2 times a month  Once a week | 27415  26059  2208 | 49.2  46.8  4.0 | 42.6  39.2  21.8 | 57.4  60.8  78.2 | 63.8  63.0  47.3 | 36.2  36.1  52.7 |
| Physical exercise  Strenuous  To some extent  A little or not at all | 12355  23148  19582 | 22.4  42.0  35.5 | 47.5  42.4  33.3 | 52.5  57.6  66.7 | 69.3  65.0  57.4 | 30.7  35.0  42.6 |
| Tooth brushing habit  Several times a day  About once a day  About 1-5-times a week or less often | 19745  25735  9985 | 35.6  46.4  18.0 | 49.2  40.0  23.3 | 50.8  60.0  76.7 | 72.1  63.2  45.8 | 27.9  36.8  54.2 |
| **Educational track** |  |  |  |  |  |  |
| Educational track  Highest  Second highest  Second lowest  Lowest | 6802  22102  20379  5617 | 12.4  40.3  37.1  10.2 | 63.4  55.4  24.8  10.1 | 36.6  44.6  75.2  89.9 | 80.1  75.3  51.2  39.7 | 19.9  24.7  48.8  60.3 |

| **Family background** |  |  |  |  |  |  |
| --- | --- | --- | --- | --- | --- | --- |
| Parents’ education  Both high  Either one high  Either one middle  Both low | 2926  6318  33337  13062 | 5.3  11.4  59.9  23.5 | 63.2  57.8  39.5  28.4 | 36.8  42.2  60.5  71.6 | 79.9  76.7  62.9  53.1 | 20.1  23.3  37.1  46.9 |
| Parents’ SES  Both upper white-collar  Either one upper white-collar  Either one lower white-collar  Either one blue-collar or both unknown | 8987  13853  17632  15210 | 16.1  24.9  31.7  27.3 | 50.3  46.9  41.2  27.1 | 49.7  53.1  58.8  72.9 | 71.9  70.0  65.0  49.6 | 28.1  30.0  35.0  50.4 |
| Family type  Nuclear  Other | 43098  12250 | 77.9  22.1 | 43.5  29.2 | 56.5  70.8 | 65.4  55.6 | 34.6  44.4 |
| **Baseline variables** |  |  |  |  |  |  |
| Gender  Male  Female | 26042  29640 | 46.8  53.2 | 46.0  54.0 | 54.0  46.0 | 54.5  70.8 | 45.5  29.2 |
| Age  12  14  16  18 | 6569  15633  17669  15811 | 11.8  28.1  31.7  28.4 | 39.2  40.2  40.3  40.6 | 60.8  59.8  59.7  59.4 | 62.7  62.5  63.4  63.9 | 37.3  37.5  36.6  36.1 |
| Survey year  1981  1985  1987  1989  1991  1993  1995  1997 | 3094  2526  8581  3065  10538  11376  8251  8251 | 5.6  4.5  15.4  5.5  18.9  20.4  14.8  14.8 | 32.1  34.4  37.5  41.0  41.0  41.6  42.0  42.9 | 67.9  65.6  62.5  59.0  59.0  58.4  58.0  57.1 | 55.5  57.0  60.4  63.0  64.4  64.9  64.8  63.0 | 44.5  43.0  39.6  37.0  35.6  35.1  35.2  37.0 |

1. The number of missing cases varies by variable.
